# Engineering of Pyroelectric Crystals Decoupled from Piezoelectricity as Illustrated by Doped α‐Glycine

**DOI:** 10.1002/anie.202213955

**Published:** 2022-11-08

**Authors:** Shiri Dishon Ben Ami, David Ehre, Andrei Ushakov, Tevie Mehlman, Alexander Brandis, Denis Alikin, Vladimir Shur, Andrei Kholkin, Meir Lahav, Igor Lubomirsky

**Affiliations:** ^1^ Department of Molecular Chemistry and Materials Science Weizmann Institute of Science Hertzel 234 Rehovot 7610001 Israel; ^2^ School of Natural Sciences and Mathematics Ural Federal University Ekaterinburg 620000 Russia; ^3^ Life Sciences Core Facilities Weizmann Institute of Science Hertzel 234 Rehovot 7610001 Israel; ^4^ CICECO-Aveiro Institute of Materials & Department of Physics University of Aveiro Aveiro 3810-193 Portugal

**Keywords:** Amino Acids, Crystal Engineering, Piezoelectricity, Pyroelectricity, Symmetry Reduction

## Abstract

Design of pyroelectric crystals decoupled from piezoelectricity is not only a topic of scientific curiosity but also demonstrates effects in principle that have the potential to be technologically advantageous. Here we report a new method for the design of such materials. Thus, the co‐doping of centrosymmetric crystals with tailor‐made guest molecules, as illustrated by the doping of α‐glycine with different amino acids (Threonine, Alanine and Serine). The polarization of those crystals displays two distinct contributions, one arising from the difference in dipole moments between guest and host and the other from the displacement of host molecules from their symmetry‐related positions. These contributions exhibit different temperature dependences and response to mechanical deformation. Thus, providing a *proof of concept* for the ability to design pyroelectric materials with reduced piezoelectric coefficient (*d*_22_) to a minimal value, below the resolution limit of the method (<0.005 pm/V).

## Graphical abstract

From symmetry considerations, a pyroelectric crystal is expected to be piezoelectric at least in its polar direction. This work demonstrates a synthetic method based on enantio‐specific co‐doping of centrosymmetric crystals. By choosing an appropriate co‐dopant with coincident contribution to pyroelectricity and opposing contributions to piezoelectricity, one can enhance the first while suppressing the second, thus breaking the linkage between the effects.

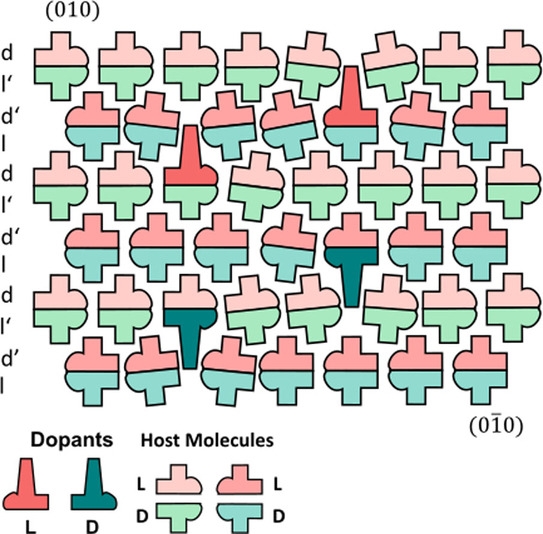

The physical and chemical properties of crystals are dictated by their symmetry.^[1]^ Specifically, out of a total 32 crystallographic classes, only the 10 polar ones might display a change in spontaneous polarization upon exposure to a thermal fluctuation. While the absolute value of polarization is not directly assessable, it's derivatives with respect to external parameters are measurable materials constants: the pyroelectric coefficient (*p*) is the derivative of the polarization (*P*) with respect to temperature (*T*), p=dP/dT (Figure 1a),^[2]^ and the piezoelectric coefficient (*d*) is the derivative of the polarization with respect to mechanical stress (*S*), d=dP/dS (Figure 1b).[^2a^, ^3^] After the discovery of piezoelectricity by the Curie brothers,[^1^, ^4^] it was demonstrated that the pyroelectricity of polar materials has two contributions:^[5]^ primary pyroelectricity arising directly from the anharmonicity of chemical bonds, leading to changes of the relative positions of the atoms without changing the overall volume of the crystal. But anharmonicity of the chemical bonds also leads to thermal expansion or contraction and therefore to a piezoelectric contribution to pyroelectricity,^[2b]^ called “secondary” pyroelectricity. With both primary and secondary contributions rooted in the common origin of chemical bond anharmonicity, in mono component polar crystals, both contributions are always present along the same polar direction, and the secondary effect is often quite comparable to the primary one.^[2b]^

**Figure 1:**
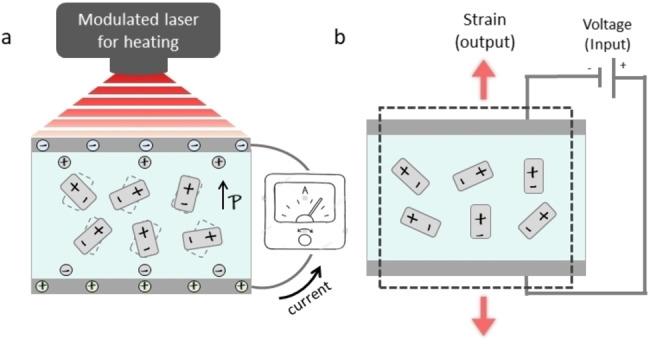
a) Pyroelectricity scheme of a single crystal as measured by periodic temperature change technique. b) Inverse piezoelectricity measurement scheme of a single crystal.

Pyroelectric and piezoelectric effects provide the backbone for a wide range of technologies, from sensors and actuators to energy harvesters.^[6]^ Therefore, devices exploiting pyroelectricity are affected by unavoidable piezoelectric effects, which provides a source of electric noise due to externally‐applied mechanic vibrations, interfering with its main operation.^[7]^ For that reason, the design of pyroelectric crystals decoupled from piezoelectricity is not only a topic of scientific curiosity but also have the potential to be advantageous. In consequence, efficient design of pyroelectric crystals with no or minimal piezoelectricity, at least along their polar crystallographic directions provides an *ongoing challenging quest*.^[8]^

Existence of such materials does not violate the Neumann's principle,^[9]^ according to which, any physical property of a crystal cannot have a symmetry lower than the crystallographic symmetry of the crystal. However, Neumann's principle does not mandate existence of any symmetry‐permitted property.

Solid solutions offer exceptional opportunities for tailoring the physical and chemical properties of materials. Consequently, the present approach is to explore the design of polar crystals by doping centrosymmetric molecular crystals, with chiral “tailor‐made” dopants.^[10]^ Such mixed crystals composed from more than one polar component provide a systematic route for a potential design of crystals, where the pyroelectricity can be decoupled from the piezoelectricity. The feasibility of this approach is illustrated here by the doping of the centrosymmetric crystals of α‐glycine with three amino acids.

**Doping α‐glycine crystals**. The α‐glycine polymorph, monoclinic, space group *P2_1_/n* ^[11]^ is centrosymmetric with four molecules in the unit cell (Figure S1 and S2). The achiral glycine molecules, assume a chiral configuration in the crystal and are organized in two pairs of chiral layers *l* and *l*′, and *d* and *d*′ (Figure 2a). Glycine molecules within the *l* or *l′* layers of the crystal are defined as *L*‐glycine and those residing within the *d* and *d′* layers as *D*‐glycine. Upon crystal growth in the presence of different amino acids as dopants, the *L*‐amino acids interact enantiospecifically with glycine molecules residing either within the *d* or the *d′* layers, which are exposed during growth at the $\left(0\bar{1}0\right)$ face. Eventually, those dopants are occluded, in a polar mode, within the *l* or *l′* layers. By symmetry considerations, *D*‐amino acid can be recognized at the $\left(010\right)$ face when either *l* or *l*′ glycine layers are exposed at this face and consequently occluded within the *d* and *d′* layers. Such enantio‐specific doping reduces the crystal symmetry to monoclinic polar space group, *P*2_1_, with the polarization along b ‐axis. (Figure 2 and S2).[^10b^, ^10d^, ^12^]

**Figure 2:**
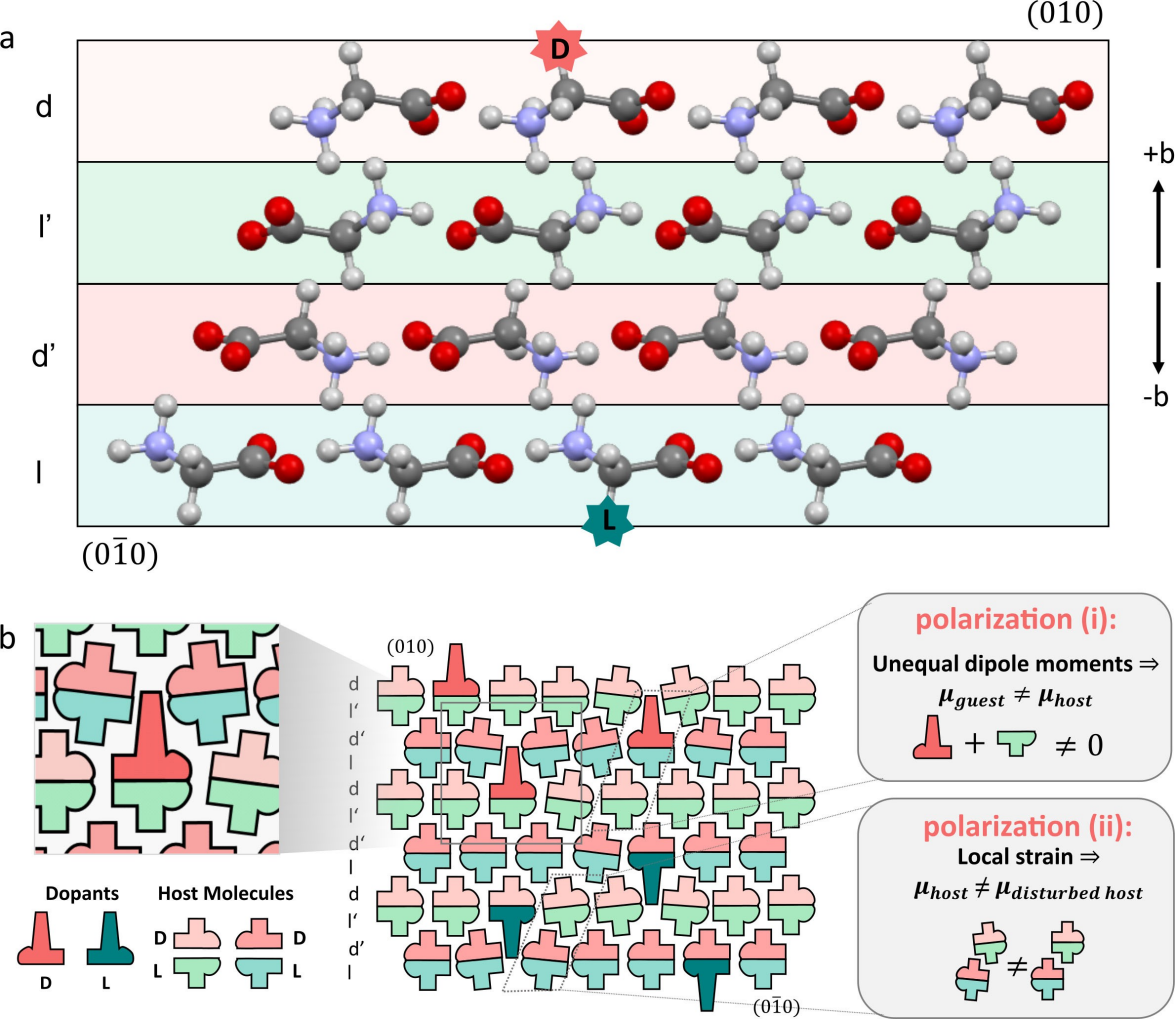
a) Enantio‐specific zwitter‐ionic interactions of *D*‐amino acids interacting with *L*‐glycine molecules. By symmetry, the *L*‐amino acids interact with *D*‐glycine molecules when the *l* and *l*′ layers are exposed at face $\left(0\bar{1}0\right)$ of the crystal. b) Two different contributions to the macroscopic polarization of the doped crystal, induced by the guest molecules: (i) Difference in dipole moments between guest amino acid and host glycine molecules. (ii) Polarization of the dislocated host glycine molecules in the neighborhood of the occluded guest amino acid.

The macroscopic polarization of such a crystal arises from two distinct structural microscopic contributions (Figure 2b). (i) The dipole moment of the guests may differ significantly from that of the host; (ii) incorporation of a guest molecule induces dislocation of neighboring host molecules in a polar mode, away from their symmetry‐related positions, relocating the dipoles out of mutual cancellation.^[13]^ Depending on the dopant, the two polarization contributions may exhibit a profoundly different temperature dependence and response to mechanical deformation. In addition, both polarizations can be further tuned by selecting guests of opposite handedness, which should be occluded from the two opposite enantiotopic $\left(010\right)$ or $\left(0\bar{1}0\right)$ faces.

Here, we offer a systematic approach for the design of mixed pyroelectric crystals with different combinations of dopants that exhibit none or minimal piezoelectricity. This is illustrated by the enantio‐specific introduction of dopants of *L*‐Threonine (*L*‐Thr), *L* and *D*‐Alanine (*L*‐ and *D*‐Ala) and *L*‐Serine (*L‐*Ser) within the centrosymmetric polymorph of α‐glycine. Each doped crystal displays pyroelectricity and piezoelectricity in different directions and exhibits also different temperature dependence of these properties. The structures of the dopants used in the study, were previously determined by DFT and M.D simulations and are shown in Figure 3.^[13]^

**Figure 3:**
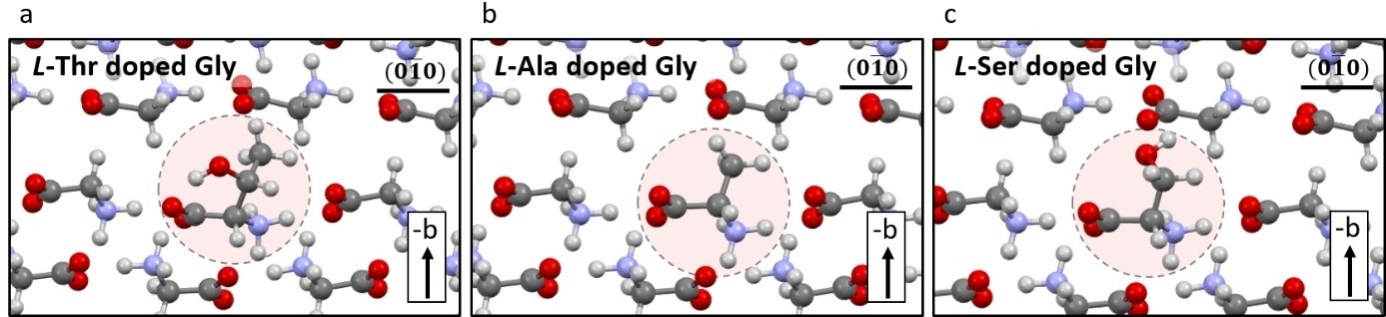
Molecular structure of doped α‐glycine. Incorporation of the guest a) *L*‐Thr, b) *L*‐Ala and c) *L*‐Ser molecules inside α‐glycine creates polar domains.^[13]^

**Pyroelectricity of α‐glycine crystals doped with** ***L*** **‐Thr,** ***L*** **‐Ala or** ***L*** **‐Ser dopants**. The pyroelectric current was measured using the periodic temperature change method^[14]^ along the *b*‐direction of α‐glycine crystals doped with *L*‐Ala, *L*‐Thr and *L*‐Ser, at 25 °C. The pyroelectric coefficient is negative for both *L*‐Thr‐doped (0.5±0.1 % mol) crystals ($p_{L-Thr}=-0.24\pm 0.06\frac{\mu C}{m^{2}K}\;$ ) (Figure 4a), and *L*‐Ala‐doped (0.5±0.2 % mol) crystals ($p_{L-Ala}=-0.11\pm 0.03\frac{\mu C}{m^{2}K}\;)$ (Figure 4b), and positive for *L*‐Ser doped crystals ($p_{L-Ser}=0.11\pm 0.03\frac{\mu C}{m^{2}K})$ (Figure 4c). All pyroelectric responses agrees with results of previous reports.^[13]^ The temperature dependence of the pyroelectric response for these doped crystals is very different. The absolute value of $p_{L-Ala}$ decreases moderately in the temperature range 23°–100 °C, while the absolute value of $p_{L-Thr}$ increases upon heating (Table 1, Figure 4a,b). The pyroelectric response of glycine doped with *L*‐Ser (Figure 4c) is different from the response of glycine doped with *L*‐Ala or *L*‐Thr. The pyroelectric coefficient of *L*‐Ser doped crystals is positive at low temperatures and become negative at higher temperatures (>40 °C). This behavior was previously explained theoretically.^[13]^ The doping of the α‐glycine crystals with the different *L*‐amino acids induces a reduction in symmetry from *P*2_1_/n to *P*2_1_, and no detectable pyroelectric effect in a ‐ and c ‐ directions was found.

**Figure 4:**
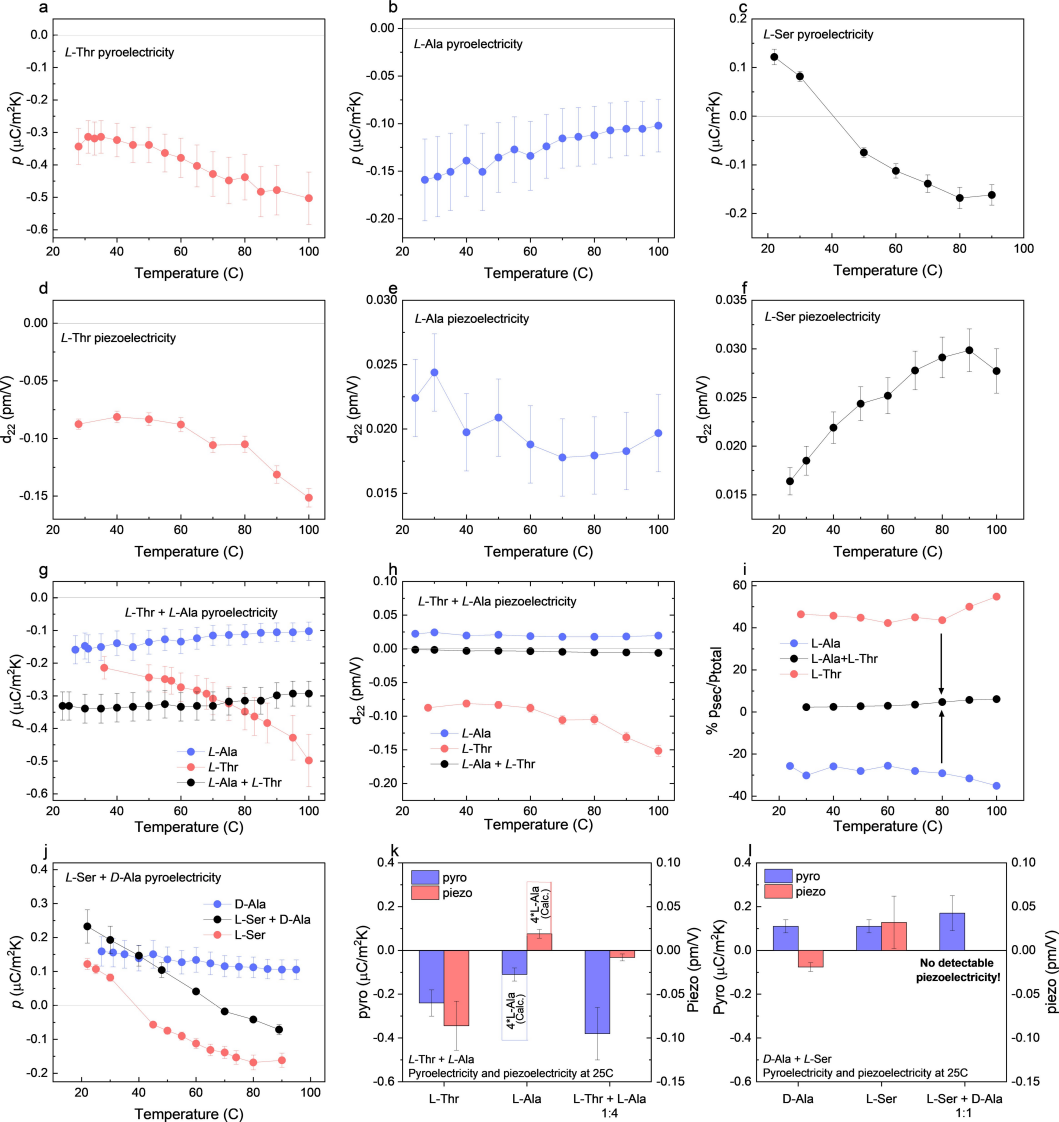
Pyroelectricity and piezoelectricity of doped α‐glycine: First row: pyroelectric response as a function of temperature of typical crystal of α‐glycine doped with a) *L*‐Thr b) *L*‐Ala c) *L*‐Ser. Second row: piezoelectric response as a function of temperature of typical crystal of α‐glycine doped with d) *L*‐Thr e) *L*‐Ala f) *L*‐Ser. Third row: g) Pyroelectric response as a function of temperature of typical crystals of *L*‐Ala, *L*‐Thr and *L*‐Ala+*L*‐Thr doped α‐glycine. h) Piezoelectric response as a function of temperature of typical crystals of *L*‐Ala, *L*‐Thr and *L*‐Ala+*L*‐Thr doped α‐glycine. i) The relative part of the secondary contribution to the pyroelectric effect from the total pyroelectricity, calculated from the data on (g) and (h). j) Pyroelectric response as a function of temperature of typical crystals of *L‐*Ala, *L*‐Ser and *L*‐Ser+*D*‐Ala doped α‐glycine. k–l) Pyroelectric and piezoelectric responses of typical crystals at 25 °C. Error bars represent the standard deviation of repeating measurements on the same crystal.

**Table 1:** Experimental values of the pyroelectric and piezoelectric responses of α‐glycine doped with various dopants.

Guest	p [μC/m^2^K]		*d*_22_ [pm/V]	
	25 °C	100 °C	25 °C	100 °C
***L*** **‐Threonine**	−0.24±0.06	−0.44±0.11	−0.086±0.028	−0.129±0.058
***L*** **‐Alanine**	−0.11±0.03	−0.08±0.02	0.019±0.005	0.014±0.009
***L*** **‐Serine**	0.11±0.03	−0.13±0.05	0.032±0.030	0.039±0.021
***L*** **‐Ala+*L*‐Thr**	−0.38±0.12	−0.30±0.14	−0.008±0.005	−0.017±0.010
***L*** **‐Ser+*D*‐Ala**	0.17±0.08	−0.09±0.02	No Detectable Piezoelectricity	

According to X‐ray diffraction (XRD), the change of the d‐spacing and the growth orientation of α‐glycine is not detectable when doping with either *L*‐Ala, *L*‐Thr or *L*‐Ser or co‐doping in the concentration 0.5–1.6 % mol.

**Piezoelectricity of α‐glycine crystals doped with** ***L*** **‐Thr,** ***L*** **‐Ala or** ***L*** **‐Ser dopants**. Michelson‐Morley laser interferometer^[15]^ was used to determine the piezoelectric strain in the *b*‐axis of the crystals$,{\;d}_{22}$ , which coincides with the same direction of the pyroelectric response. We found that the responses, at 25 °C, of the crystals doped with *L*‐Ala and *L*‐Ser have opposite signs than crystals doped with *L*‐Thr (Figure 4k): for *L*‐Thr $d_{22L-Thr}=-0.086\pm 0.028\;\;pm/V$ (Figure 4d), while for *L*‐Ala doped crystals $d_{22L-Ala}=0.019\;\pm 0.005\;\;pm/V$ (Figure 4e) and for *L*‐Ser doped crystals $d_{22L-Ser}=0.032\;\pm 0.030\;\;pm/V$ (Figure 4f). While for *L*‐Ser doped crystals, the pyroelectric response changes its direction with increasing the temperature, the piezoelectric response stays in the same direction for all the temperature range measured (23°–100 °C) (Figure 4f). For *L*‐Ala and *L*‐Thr doped crystals the piezoelectric response preserved the same direction at all temperature range measured (23°–100 °C).

**Pyroelectricity and piezoelectricity of co‐doped α‐glycine crystals**. According to the measured directions of the pyroelectric and piezoelectric responses of *L*‐Ala, *L*‐Ser and‐ *L*‐Thr doped glycine crystals, we anticipated that co‐doping with appropriate concentrations of *L*‐Ala+*L*‐Thr and *D*‐Ala+*L*‐Ser should yield crystals that exhibit no piezoelectricity while enhancing their pyroelectricity along its polar *b‐*axis. (Figure 4k).

Indeed, at least two out of ten crystals with the composition *L*‐Thr:*L*‐Ala≈1 : 4 (0.07±0.02 % mol *vs*. 0.3±0.1 % mol) showed no detectable piezoelectricity (or at least under the resolution limit of the interferometer (<0.005 pm/V)). In the other crystals, the piezoelectric effect in the polar direction is near the resolution limit of the interferometer with an average of $d_{22L-Ala+L-Thr}=-0.008\;\pm 0.004\frac{pm}{V},$ which increases very slightly between 23 °C and 100 °C and shows no dependence in the frequency in the range 2–20 kHz (Figure 4h,k and S3, Table 1 **)**. At the same time, the co‐doped crystal preserves the pyroelectric effect, $p_{L-Thr+L-Ala}=-0.38\pm 0.13\frac{\mu C}{m^{2}K}$ (Figure 4g and S3). The pyroelectric response is negative for both *L*‐Ala and *L*‐Thr doping, and since *L*‐Ala is present in a larger concentration than *L*‐Thr, the absolute value of the pyroelectric response decreases with temperature, similar to *L*‐Ala‐doped glycine (Figure 4g). From the pyroelectric and piezoelectric responses for the co‐doped crystal, the thermal expansion response of α‐glycine, $\beta_{glycine}=7\cdot 10^{-5}\;K^{-1}$ ,^[13]^ and its Young modulus in the *b*‐direction, $Y_{glycine}=26\;\;GPa$ ,^[16]^ one can see that the secondary contribution to the pyroelectricity of the co‐doped crystal (Figure 4i**)** to be negligibly small with respect to the primary one:

(1) $$p_{co-doped\_sec}=d_{22\_co-doped}\cdot \beta_{glycine}\cdot Y_{glycine}\ll p_{co-doped\_prim}$$

One of the advantages of the centrosymmetric crystals is that they are contains pairs of enantiotopic faces. Dopants of opposite chiralities should be occluded within the host crystals from opposite faces. Therefore, doping crystal with *D*‐enantiomer will induce polarization in opposite direction in comparison to the *L*‐enantiomer and the pyroelectric and the piezoelectric responses will be inverted. For obtaining such crystals we used pure α‐glycine seed crystals glued on their $\left(010\right)$ face at the bottom of the crystallization dish (Figure 5). Consequently, both faces, $\left(010\right)$ and $\left(0\bar{1}0\right)$ , through which the *D* and *L* enantiomers are occluded, are exposed to the solution.

**Figure 5:**
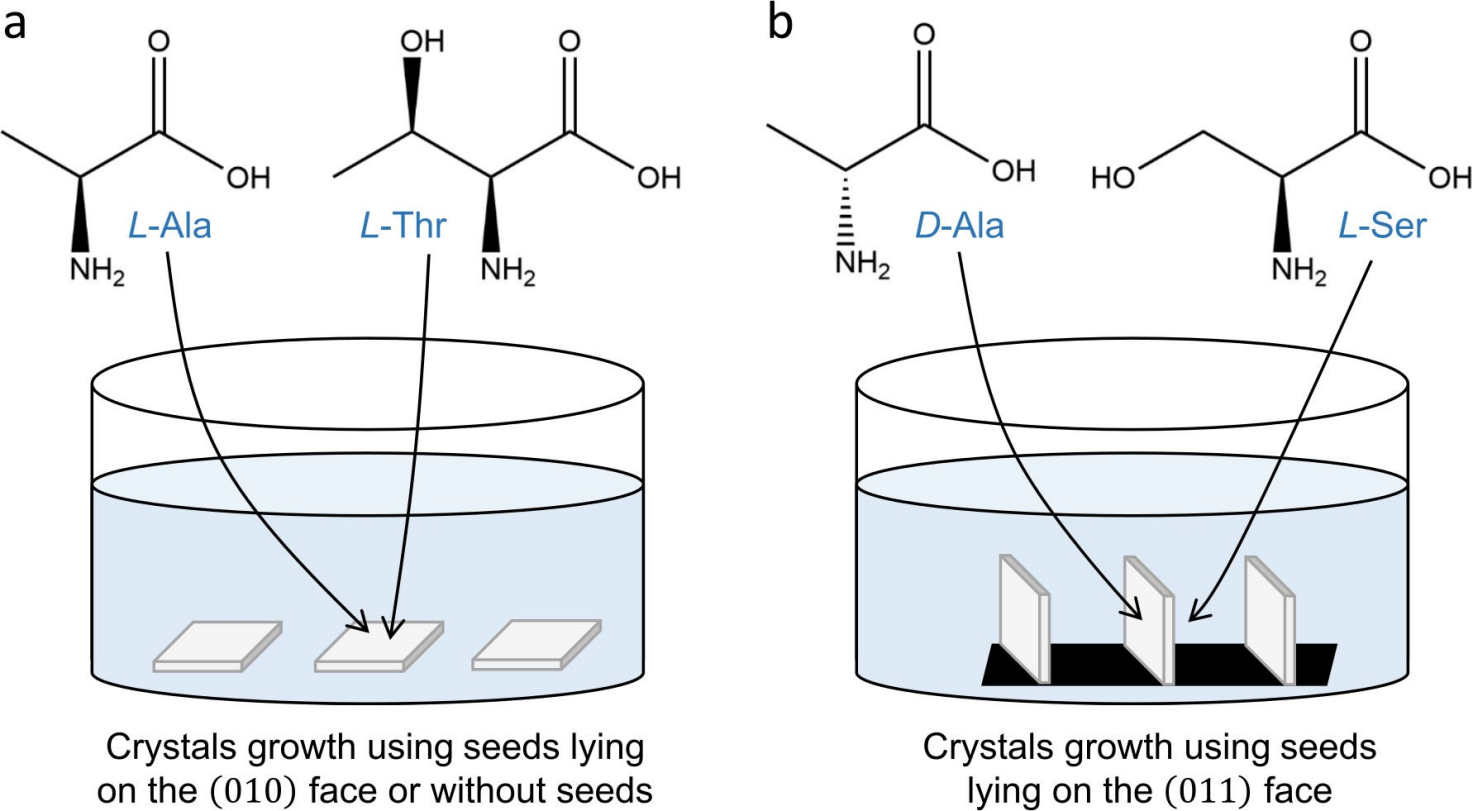
a) Glycine crystals grown without seeds lying on the $\left(010\right)$ face exposing its enantiotopic $\left(0\bar{1}0\right)$ face to the solution as ‐doped with *L*‐amino acids. b) Growth method of crystals doped both with *D‐*Ala and *L‐*Ser. The $\left(011\right)$ faces of the pure α‐glycine seeds were glued with carbon tape to the bottom of the crystallization dish, thus exposing both $\left(010\right)$ and $\left(0\bar{1}0\right)$ faces to the solution during crystal growth.

*L*‐Ser and *D*‐Ala were used as co‐dopants based on their piezoelectric and pyroelectric properties as measured for each dopant separately. The *D‐*Ala is occluded from the $\left(010\right)$ face and the *L*‐Ser from the $\left(0\bar{1}0\right)$ face. (Figure 2, Figure 5).

*L*‐Ser doped crystals has piezoelectric coefficient of $d_{22}=0.032\;\pm 0.030$ pm/V at 25 °C, in opposite direction to *D*‐Ala, which will incorporate into the crystal from the opposite crystallographic direction to the *L*‐Ser. The combination of the two dopants is anticipated to decrease the piezoelectric response. Indeed, in crystals with the composition *L*‐Ser:*D*‐Ala≈1 : 1 (0.9±0.3 % mol vs. 0.8±0.1 % mol) the piezoelectric response is suppressed to a value under the resolution limit of the interferometer (<0.005 pm/V). In contrast to the suppressing the piezoelectric response, the pyroelectric coefficient of the co‐doped crystal with *L*‐Ser and *D*‐Ala is larger than the coefficient of the crystals doped with *L*‐Ser ($p_{L-Ser+D-Ala}=0.17\pm 0.08\frac{\mu C}{m^{2}K}$ at 25 °C) (Table 1 and Figure 4j) and the temperature in which the coefficient become negative shifts to higher temperature because the positive contribution of the *D*‐Ala to the pyroelectric response.

The other than $d_{22}$ components (see supplementary text) of the piezoelectric tensor of the crystals could not be measured with the interferometer due to thin truncated pyramid shape of the crystals (Figure S1). Therefore, their lateral (shear) piezoelectric response was probed with the piezo‐response force microscopy (PFM).^[17]^ Similar to the case of ${\;d}_{22}$ , the shear piezoelectric response of the co‐doped crystal, <0.01 pm/V, is in the detection limit, smaller than that of the *L*‐Thr and *L*‐Ala doped glycine crystals. This indicates that, the proposed co‐doping method may suppress the piezoelectric tensor in more than one direction at a wide temperature range (bellow 0.005 pm/V).

The synthetic method reported here describes the conversion of centrosymmetric crystals into pyroelectric ones. Since those crystals are delineated by pairs of enantitiopic faces, by choosing “tailor made” chiral dopants either of the same or of opposite handedness, one can control the direction of the polarization they induce.

Although the pyroelectric coefficient of α‐glycine is too low for immediate practical applications (commercial pyroelectric materials show pyroelectric response of 100 s μC/m^2^K), the doped α‐glycine with different α‐amino acids were selected in this proof‐of‐concept study for the following advantages: the zwitterions of the amino acids have a large dipole moment (14.9 D for glycine).^[18]^ Therefore, even this small concentration of dopant induces well‐measurable pyroelectric and piezoelectric effects.[^10d^, ^13^] In addition large single crystals can be easily grown and the dopant‐induced local distortions were calculated in great details.^[13]^ Finally the amino acids besides proline, contains the same zwitterionic group, as present in the glycine molecules, provides an abundant assembly of appropriate dopants of opposite handedness that should provide a variety of systems.

Those mixed crystals, contain at least two contributions to polarization: (i) induced by the difference in dipole between the dopant and the host molecule it replaces, and (ii) induced by the distorted neighboring molecules of the dopants (Figure 2b). Therefore, major contributions to the pyroelectric and piezoelectric effects may arise from different components of the crystal polarization. Accordingly, they may have different directions and temperature dependences. By choosing the appropriate combination of dopants, the nearly universal linkage between pyroelectricity and piezoelectricity, can be severed as demonstrated here.

This work provides a *proof‐of‐concept* showing that considering polar incorporation of “tailor‐made” co‐dopants into centrosymmetric molecular crystals allow the design of host–guest system where the piezoelectric and pyroelectric responses decoupled along a temperature range. By adjusting the concentration, and handedness of the dopants one can reduce significantly and even may reach a suppression of the piezoelectricity while preserving or even enhancing the pyroelectricity.

Although the quantities of dopants used in this work are minor, using dopants that are smaller in size than the host molecules will allow significant increase of the dopant concentration. Thus, the proposed material design concept has the potential to be expended to large variety of mixed molecular crystals, and quasi‐racemates, and may become a valuable addition to the materials design toolbox, enabling the materials engineering of with previously unattainable combinations of functional properties.

## Conflict of interest

The authors declare no conflict of interest.

## Supporting information

As a service to our authors and readers, this journal provides supporting information supplied by the authors. Such materials are peer reviewed and may be re‐organized for online delivery, but are not copy‐edited or typeset. Technical support issues arising from supporting information (other than missing files) should be addressed to the authors.

Supporting Information

## Data Availability

The data that support the findings of this study are available from the corresponding author upon reasonable request.
