# Supplementary material for: Engineering of Pyroelectric Crystals Decoupled from Piezoelectricity as Illustrated by Doped α‐Glycine
Source: Angew Chem Int Ed Engl. 2022 Nov 8;61(49):e202213955. doi: 10.1002/anie.202213955 (PMC10100455; doi:10.1002/anie.202213955)
Supplement: Supplementary file 1 — Supporting Information [file ANIE-61-0-s001.pdf]

Angewandte Chemie
Eine Zeitschrift der Gesellschaft Deutscher Chemiker

Supporting Information

Engineering of Pyroelectric Crystals Decoupled from Piezoelectricity as Illustrated by Doped α-Glycine

S. Dishon Ben Ami, D. Ehre, A. Ushakov, T. Mehlman, A. Brandis, D. Alikin, V. Shur, A. Kholkin, M. Lahav*, I. Lubomirsky**

Supporting Information
©Wiley-VCH 2021
69451 Weinheim, Germany

Engineering of Doped Pyroelectric Crystals Decoupled from Piezoelectricity

Shiri Dishon Ben Ami,[a] David Ehre,[a] Andrei Ushakov,[b] Tevie Mehlman,[c] Alexander Brandis,[c] Denis Alikin,[d] Vladimir Shur,[b] Andrei Kholkin,* [d] Meir Lahav,*[a] and Igor Lubomirsky*[a]

Abstract: Symmetry dictates that the 10 polar out of the 32 crystal classes are expected to be pyroelectric and piezoelectric. Design of pyroelectric crystals decoupled from piezoelectricity is not only a topic of scientific curiosity but is also technologically advantageous. Here we report a new approach for the design of such materials. The method comprises the co-doping of centrosymmetric crystals with tailor-made guest molecules, as illustrated by the doping of α-glycine polymorph with different amino acids (Threonine, Alanine and Serine). The polarization of those crystals displays two distinct contributions, one arising from the difference in dipole moments between guest and host and the other from the displacement of host molecules from their symmetry-related positions. These contributions exhibit different temperature dependences and response to mechanical deformation. Thus, providing a proof of concept for the ability to design pyroelectric materials with no or minimal piezoelectricity.

DOI: 10.1002/anie.2021XXXXX

Table of Contents

Experimental Procedures 2
Supplementary Text 4
Supplementary Figures 5
Supplementary Tables 9
References 9
Author Contributions 9

Experimental Procedures

Crystal Growth

Supersaturated solution (130%) of glycine (Alfa Aesar 99.5+%) with 5%wt^{-1} dopants was prepared by dissolving the amino acid in water (ultra-pure Millipore water, 18.2 MΩ cm at 25°C, Millipore Synergy UV, Type 1 water) and heating to 80°C to achieve complete dissolution. The solutions filtered through cotton wool into glass crystallization dishes, which were covered with filtration papers for allowing slow evaporation in a cleanroom environment. The transparent single crystals were washed in water, dried, and heated to 100 °C for 24 hours to remove surface polarization induced by the incorporation of water at the surfaces of the crystals. The doped crystals were grown in presence of: 5%wt^{-1} *L*-alanine (Chem-Impex Int'l Inc., 99.2%), *L*-threonine (Fisher Scientific, 99.0-101.0%) and *L*-Serine (Sigma Aldrich, >99% (TLC)). The *L*-Alanine and *L*-Threonine co-doped crystals were grown in the presence of 3.3%wt^{-1} *L*-alanine and 1.67%wt^{-1} *L*-threonine.

The *L*-Serine and *D*-Alanine co-doped crystals were grown using α-glycine seeds and in the presence of 5%wt^{-1} *L*-serine and 5%wt^{-1} *D*-alanine (Chem-Impex Int'l Inc., 99.4%). To allow the occlusion of L- and D-dopants, the seeds were glued on their (011) face, using carbon tape to the crystallization dish (**Fehler! Verweisquelle konnte nicht gefunden werden.**).

Pyroelectricity Measurements

The pyroelectric current was measured by periodic temperature change (PTC) technique as described in ref[1] (Fehler! Verweisquelle konnte nicht gefunden werden.**a**). The pyroelectric response was calculated from the measured pyroelectric current (I) using the eq (2):

$$(2)\quad p = \frac{I \cdot d \cdot C_v}{A \cdot F}$$

where d is the sample thickness, A is the area subjected to the laser illumination, $C_v = 1.545\, J/cm^3 K$ is the heat capacity of α-glycine and F is the laser heat flux.

Piezoelectricity Measurements

The piezoelectric response was measured using a Michelson-Morley interferometer equipped with lock-in amplifier (SR830, Stanford Research Systems Inc., USA)[2]. A PID feedback system based on piezoactuator P-841.01 and piezocontroller E-709.SRG (Physik Instrumente GmbH & Co. KG, Germany) was employed to correct slow optical path length drifts for working point stabilization. External voltage was applied to the membranes with an Agilent 33210A (Keysight Technologies, USA) signal generator. A customized heating stage of our own design was used (**Figure S4**). A three-layered stage consisting of two copper plates with a size of 30 × 30 × 1.5 mm^3 was clamped by an electrically insulated titanium foil which was used as the heating element. The temperature stabilization system consisting of a Pt100 class B resistance temperature sensor (Heraeus Nexensos, Germany), a MB110 software I/O module (Owen, Russia), and a ZUP60-3.5 programmable power supply (TDK-Lambda Corporation, Japan) was used. Thermal insulation of the heating stage was carried out using an insulating polyurethane foam box, installed on a platform made from polytetrafluoroethylene (PTFE), which insulated the heating element. The temperature stability was better than 0.01°C/min at 40°C and 0.1°C/min at 120 °C. The measurements were carried out with an excitation signal of 100–150 V amplitude in a frequency range 2–20 kHz which was sufficient to eliminate the interference from mechanical resonances and electrical and electromagnetic noise. Each data point was obtained at a chosen frequency by averaging at least three measurements of strain amplitude versus applied voltage. Every sample was cycled within the temperature range 25 – 120 °C for three times.

Liquid-Chromatography tandem Mass-Spectrometry (LC-MS/MS) for Compositional Characterization

The doped crystals were dissolved in DDW and analyzed by LC-MS/MS using UPLC Acquity I-class and Zevo TQ-S MS (both Waters). For the separation, BEH Amide UPLC column 1.7 um (Waters) was used with a gradient of acidified ammonium formate/acetate and acetonitrile as solvents. For mass spectrometry electrospray ionization in positive mode was used. Multiple reaction monitoring (MRM) parameters optimized for each amino acid were applied using corresponding standards. Quantification was based on standard curves of amino acid mix in concentration range 1-100uM using TargetLynx software.

Supplementary Text

In the crystal system of the doped α-glycine (monoclinic, $P_{2/1}$) there are 8 independent piezoelectric coefficients: d_{21}, d_{22}, d_{23}, d_{14}, d_{16}, d_{25}, d_{34} and d_{36}.

Supplementary Figures

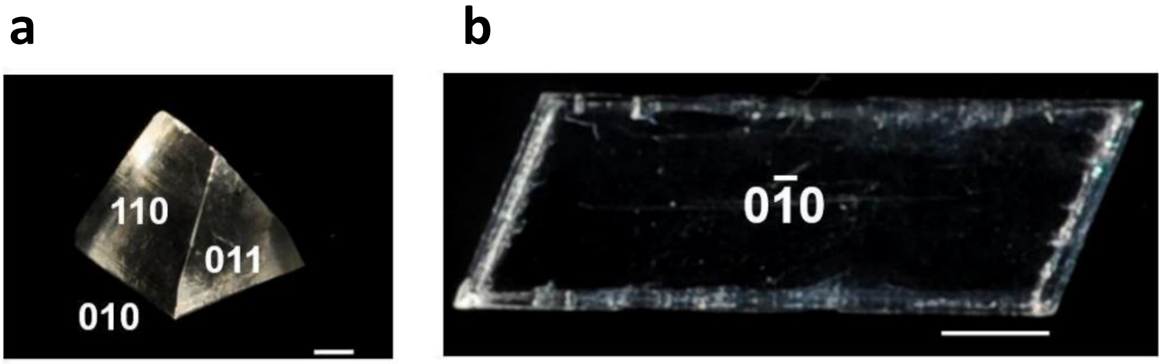

Figure S1. Photographs of the α-glycine crystals. (a) Pure α-glycine crystal. (b) α-glycine crystal doped with $\sim 0.5\ \%mol$ L-amino acid. Scale bars, 1 mm.

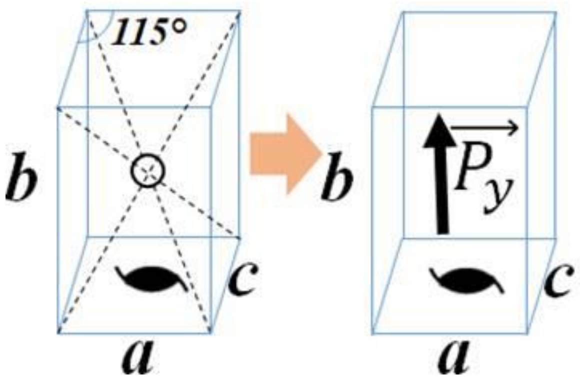

Figure S2. Symmetry reduction upon stereospecific doping α-$glycine$ crystal with L-amino acid making b-axis polar.

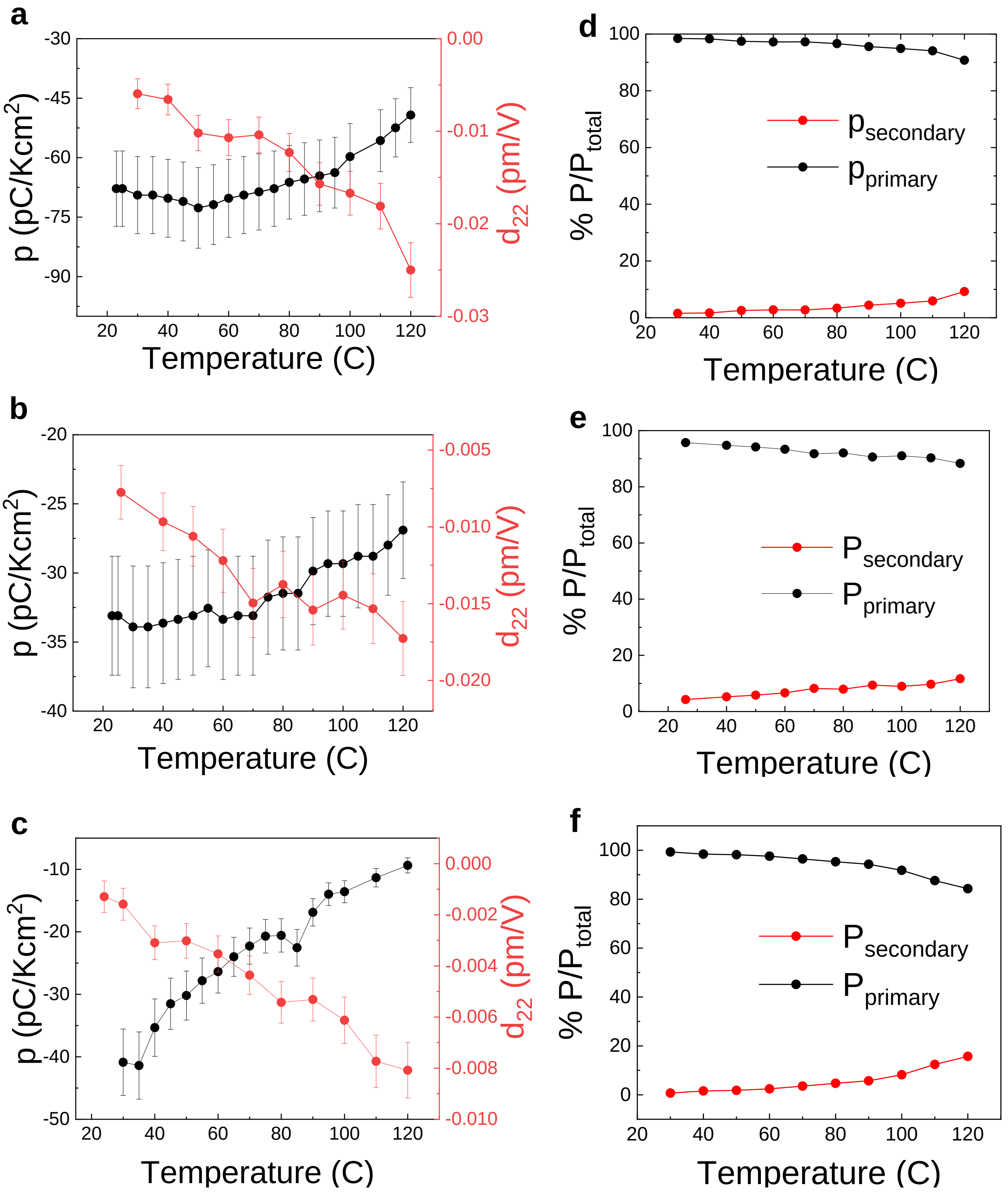

Figure S3. Pyroelectricity and piezoelectricity of α-glycine co-doped with *L*-Ala and *L*-Thr. (a-c) pyroelectric coefficient (black), p, and piezoelectric coefficient (red), d_{22}, as function of temperature for different typical crystals. (d-e) The relative part of the primary (black) and secondary (red) contributions to the total pyroelectric effect calculated from the data on (a-c), respectively. Error bars represent the standard deviation of repeating measurements on the same crystal.

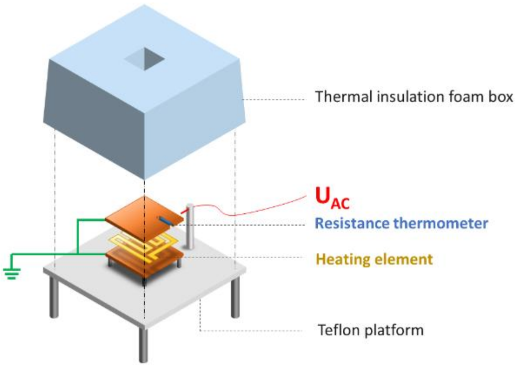

Figure S4. Schematics illustration of the customized heating stage for piezoelectricity measurements.

Supplementary Tables

Table S1. Dopants concentrations as determined by LC-MS/MS.

Dopants		Concentration [%mol]
***L*-Ala**		0.522 ± 0.178
***L*-Thr**		0.467 ± 0.128
**L-Ser**		1.572 ± 0.391
***L*-Thr + *L*-Ala**	*L*-Ala	0.286 ± 0.116
	L-Thr	0.066 ± 0.025
***L*-Ser + *D*-Ala**	*D*-Ala	0.791 ± 0.136
	L-Ser	0.920 ± 0.299

References

[1] a)E. Meirzadeh, I. Azuri, Y. Qi, D. Ehre, A. M. Rappe, M. Lahav, L. Kronik, I. Lubomirsky, *Nat Commun* **2016**, *7*; b)I. Lubomirsky, O. Stafsudd, *Rev Sci Instrum* **2012**, *83*; c)A. G. Chynoweth, *J Appl Phys* **1956**, *27*, 78-84.

[2] a)A. D. Ushakov, N. Yavo, E. Mishuk, I. Lubomirsky, V. Y. Shur, A. L. Kholkin, *Kne Mater Sci* **2016**, *2016*, 177-182; b)E. Mishuk, A. D. Ushakov, S. R. Cohen, V. Y. Shur, A. L. Kholkin, I. Lubomirsky, *Solid State Ionics* **2018**, *327*, 47-51.

Author Contributions

Conceiving and designing the experiment: IL, DE, ML, SD, AK, VS.

Sample preparation: SD.

Performance of experiments: SD, AU, TM, AB, DA

Data Analysis: IL, DE, ML, SD, AK, AU.

Writing – original draft: IL, DE, ML, SD.

Writing – review & editing: all co-authors
